# Supplementary material for: A potent human monoclonal antibody with pan-neutralizing activities directly dislocates S trimer of SARS-CoV-2 through binding both up and down forms of RBD
Source: Signal Transduct Target Ther. 2022 Apr 5;7:114. doi: 10.1038/s41392-022-00954-8 (PMC8980211; doi:10.1038/s41392-022-00954-8)
Supplement: Supplementary file 1 — Supplementary materials [file 41392_2022_954_MOESM1_ESM.docx]

Supplementary Materials for

A potent human monoclonal antibody with pan-neutralizing activities directly dislocates S trimer of SARS-CoV-2 through binding both up and down forms of RBD

Xiaofei Wang, Ao Hu, Xiangyu Chen, Yixin Zhang, Fei Yu, Shuai Yue, Arong Li, Junsong Zhang, Zhiwei Pan, Yang Yang, Yao Lin, Leiqiong Gao, Jing Zhou, Jing Zhao, Fang Li, Yaling Shi, Feng Huang, Xiaofan Yang, Yi Peng, Luoyang Tu, Huan Zhang, Huanying Zheng, Jun He, Hui Zhang, Lifan Xu, Qizhao Huang, Yongqun Zhu, Kai Deng, Lilin Ye

Correspondence to: yelilinlcmv@tmmu.edu.cn (Lilin Ye), dengkai6@mail.sysu.edu.cn (Kai Deng) and zhuyongqun@zju.edu.cn (Yongqun Zhu)

**This PDF file includes:**

Figures. S1 to S7

Table S1

**
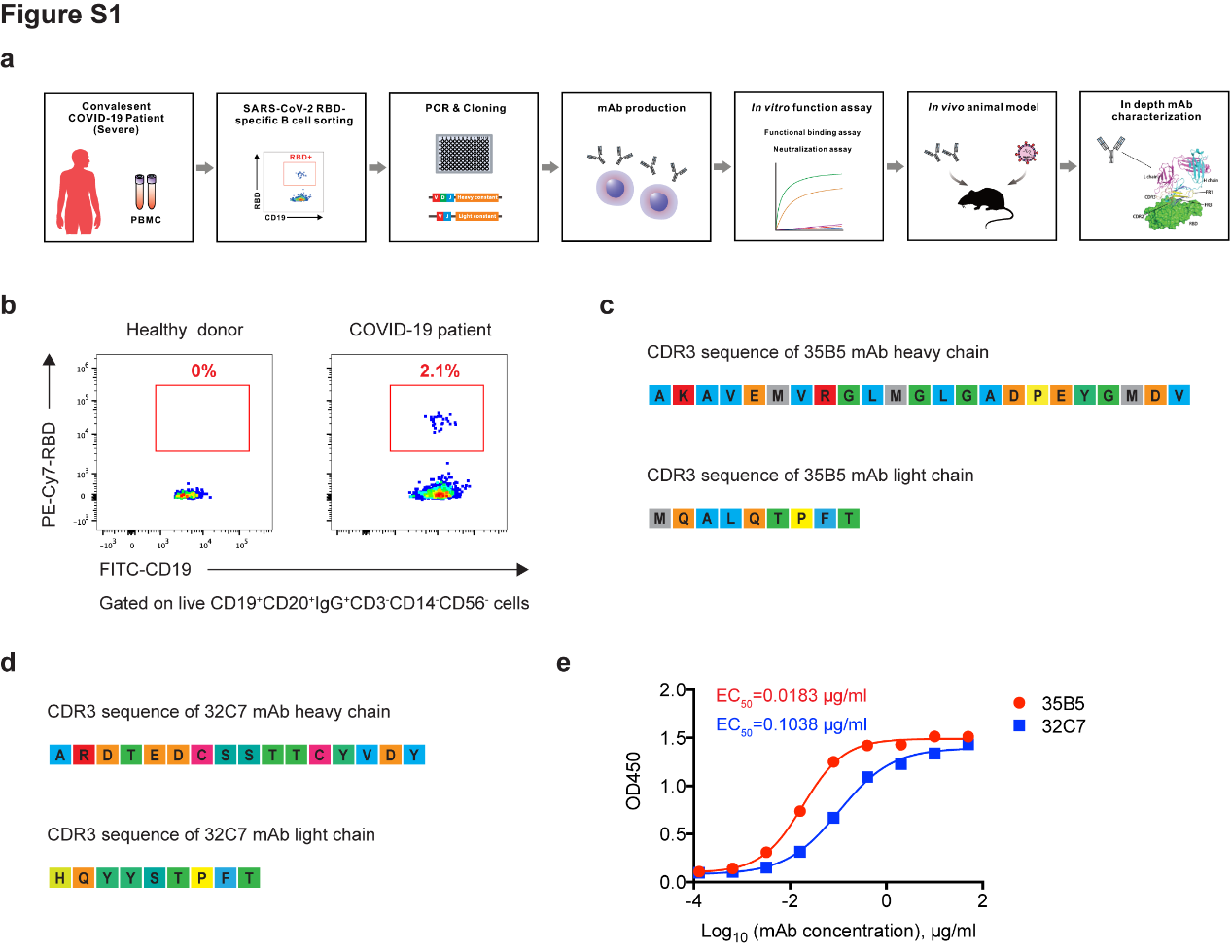
**

**Figure. S1. Isolation of potent SARS-CoV-2 neutralizing mAbs from COVID-19 convalescent patients. a** Isolation strategy of SARS-CoV-2 neutralizing mAbs. **b** Flow cytometry analysis of SARS-CoV-2 RBD-specific B cells from the PBMC of healthy donors and COVID-19 convalescent patients. The numbers adjacent to the outlined area indicate the proportions of SARS-CoV-2 RBD-specific B cells in CD19^+^CD20^+^IgG^+^ B cells. **c, d** The CDR3 sequences of mAb 35B5 (**c**) and mAb 32C7 (**d**). **e** ELISA analysis of mAb 35B5 (red) or mAb 32C7 (blue) binding to SARS-CoV-2 RBD protein. EC_50_, concentration for 50% of maximal effect.

**
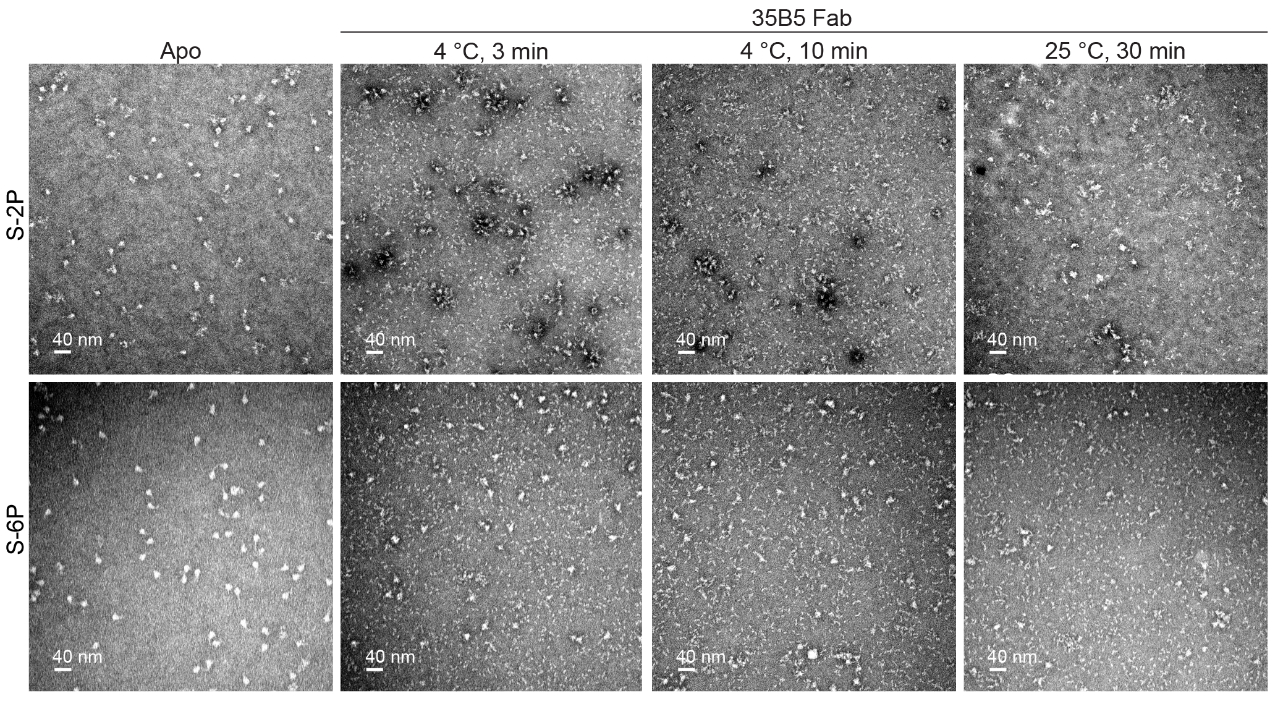
**

**Figure. S2. Representative negative-staining EM micrographs of the S-2P and S-6P trimers after the treatment of 35B5 Fab.** The S-2P and S-6P trimeric proteins were treated with or without 35B5 Fab for indicated time at 4^o^C or 25^o^C before negative staining analysis. Scale bar, 40 nm.


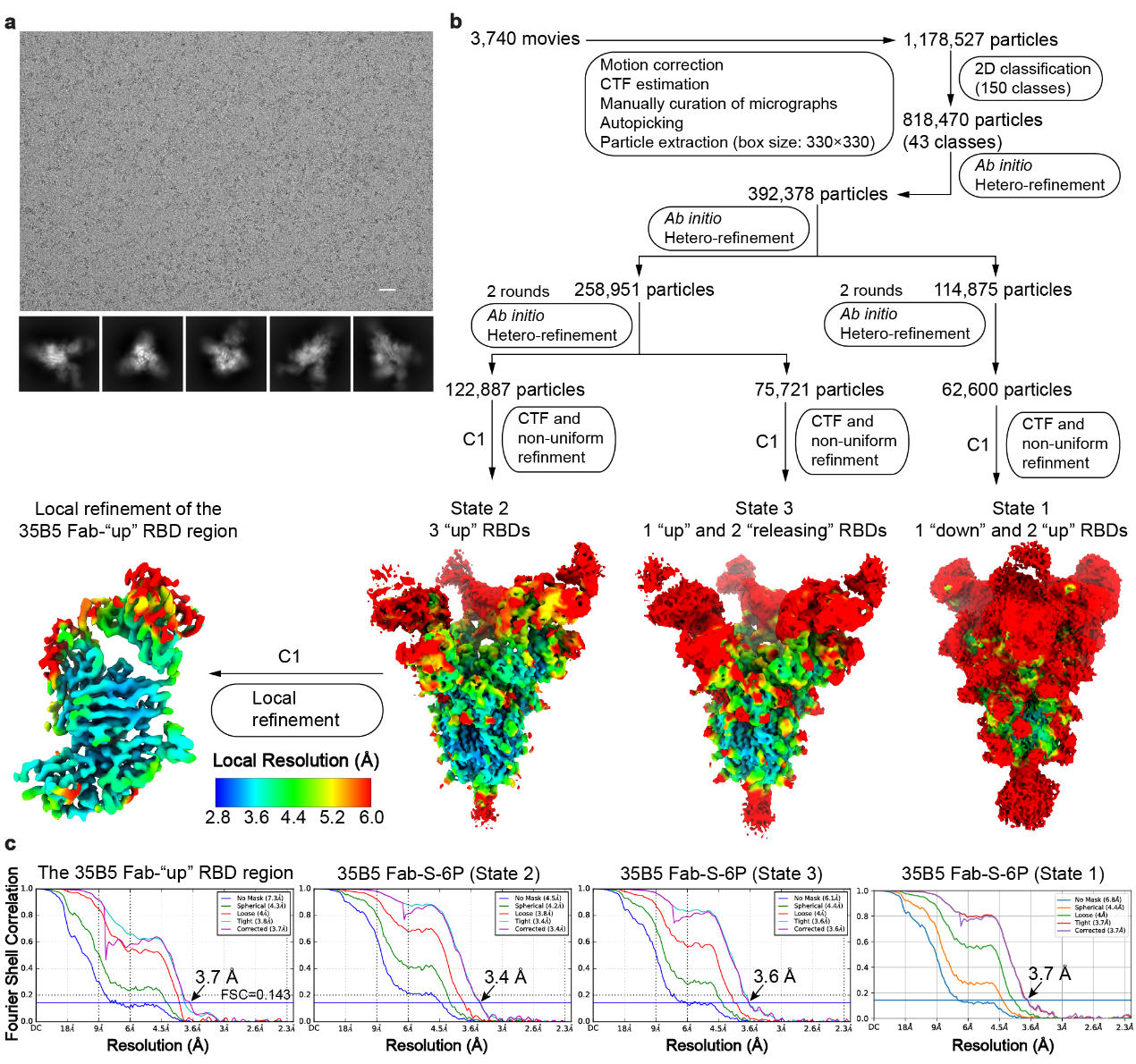


**Figure. S3. Cryo-EM data processing and validation of the S-6P-35B5 Fab complex.** **a** Representative cryo-electron micrograph (upper) and 2D class averages (lower) of the S-6P-35B5 Fab complex. Scale bar, 25 nm. **b** Cryo-EM data processing flow-chart of the S-6P-35B5 Fab complex. Three states of the S-6P-35B5 Fab complex were obtained in data collection. Local refinement of the 35B5Fab-“up” RBD region was carried out using C1 symmetry (left). All density maps were prepared using ChimeraX. The map resolution is color coded for different regions. The resolution goes from 2.8 to 6.0 Å. **c** The Fourier shell correlation (FSC) curves for the reconstructions in (**b**). The resolution estimations of cryo-EM density maps were based on the corrected FSC curves at the gold standard FSC=0.143 criterion.


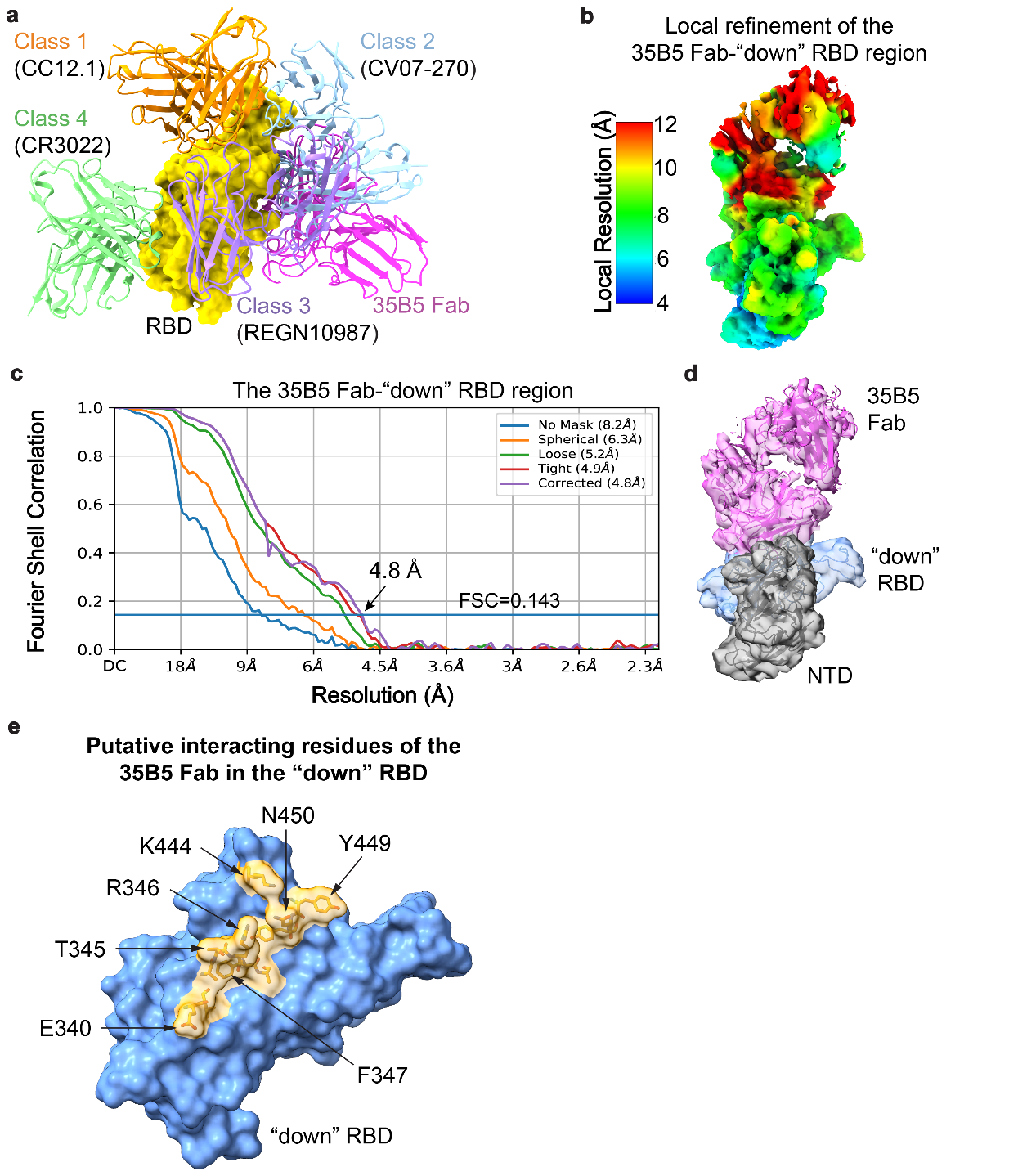


**Figure. S4. Comparison of 35B5 with other mAbs and the local refinement of 35B5 Fab-“down” RBD region. a** Structural comparisons of the 35B5 Fab-“up” RBD interactions with those of the previously identified four classes of neutralizing antibodies. 35B5 Fab and the Fab regions of the representative antibodies CC12.1, CV07-270, REGN10987 and CR3022 from Class 1-4, respectively, are illustrated in cartoon and colored as indicated. RBD is represented as surface in yellow. **b** Local refinement of the region of 35B5 Fab with the “down” RBD and NTD domains in the State 1 S-6P-35B5 Fab complex. The density map was prepared using ChimeraX. The map resolution is color coded for different regions. The resolution goes from 4.0 Å to 12 Å. **c** The Fourier shell correlation (FSC) curve for the reconstructions in (**b**). The resolution estimation (4.8 Å) of cryo-EM density maps were based on the corrected FSC curves at the gold standard FSC=0.143 criterion. **d** Structural modeling of 35B5 Fab with the “down” RBD and NTD domains in the density map obtained from the local refinement in (**b**). 35B5 Fab, RBD and NTD are colored in purple, blue and grey respectively. **e** The putative 35B5-interacting residues of the “down” RBD. The 35B5-interacting residues of the “down” RBD (within 4 Å to 35B5 Fab) are colored in yellow.


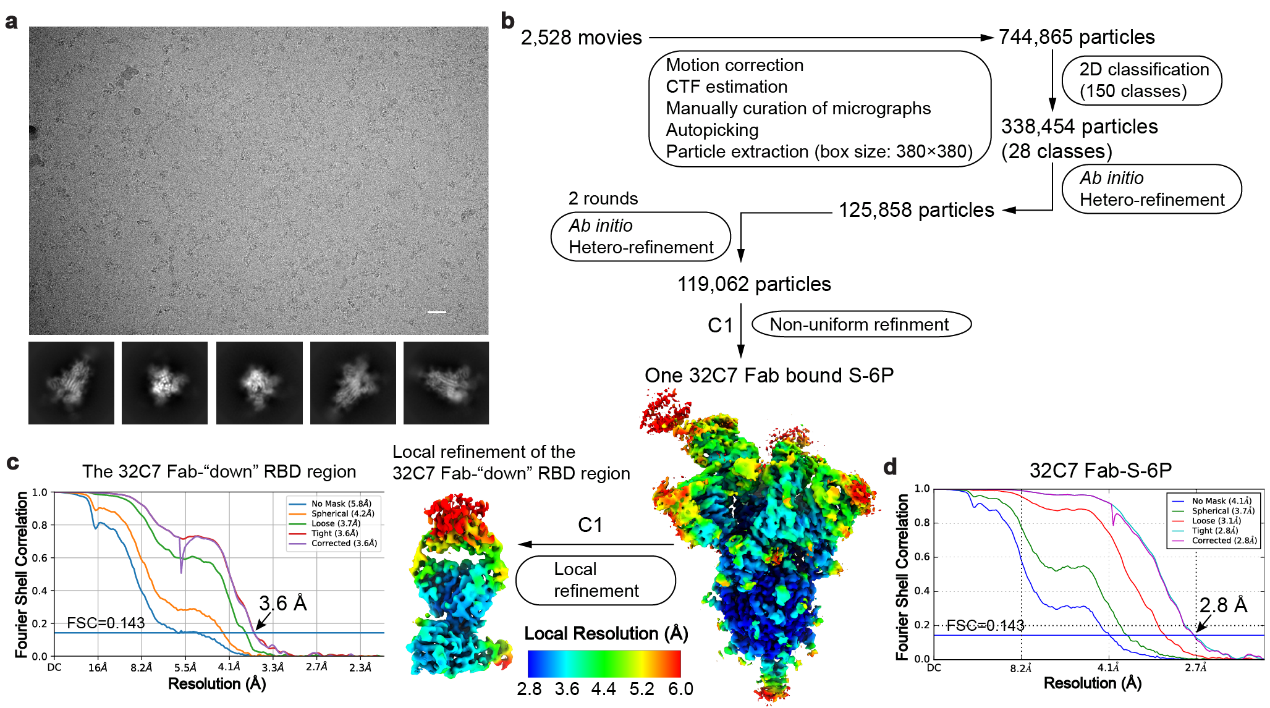


**Figure. S5. Cryo-EM data processing and validation of the S-6P-32C7 Fab complex. a** Representative electron micrograph (upper) and 2D class averages (lower) of the SARS-CoV-2 S-6P- 32C7 Fab complex. Scale bar, 25 nm. **b** The cryo-EM data processing flow chart of the S-6P-32C7 Fab complex. Only one 32C7 Fab is bound to a “down” RBD of the S-6P trimer. Local refinement of 32C7 Fab with the “down” RBD generated a map at the resolution of ~ 3.6 Å. The density map was prepared using ChimeraX. The map resolution is color coded for different regions. The resolution goes from 2.8 Å to 6.0 Å. **c**, **d** The FSC curves for the reconstructions of the 32C7 Fab-RBD region (**c**) and the S-6P-32C7 Fab complex (**d**) in (**b**). The resolution estimations of cryo-EM density maps were based on the corrected FSC curves at the gold standard FSC=0.143 criterion.


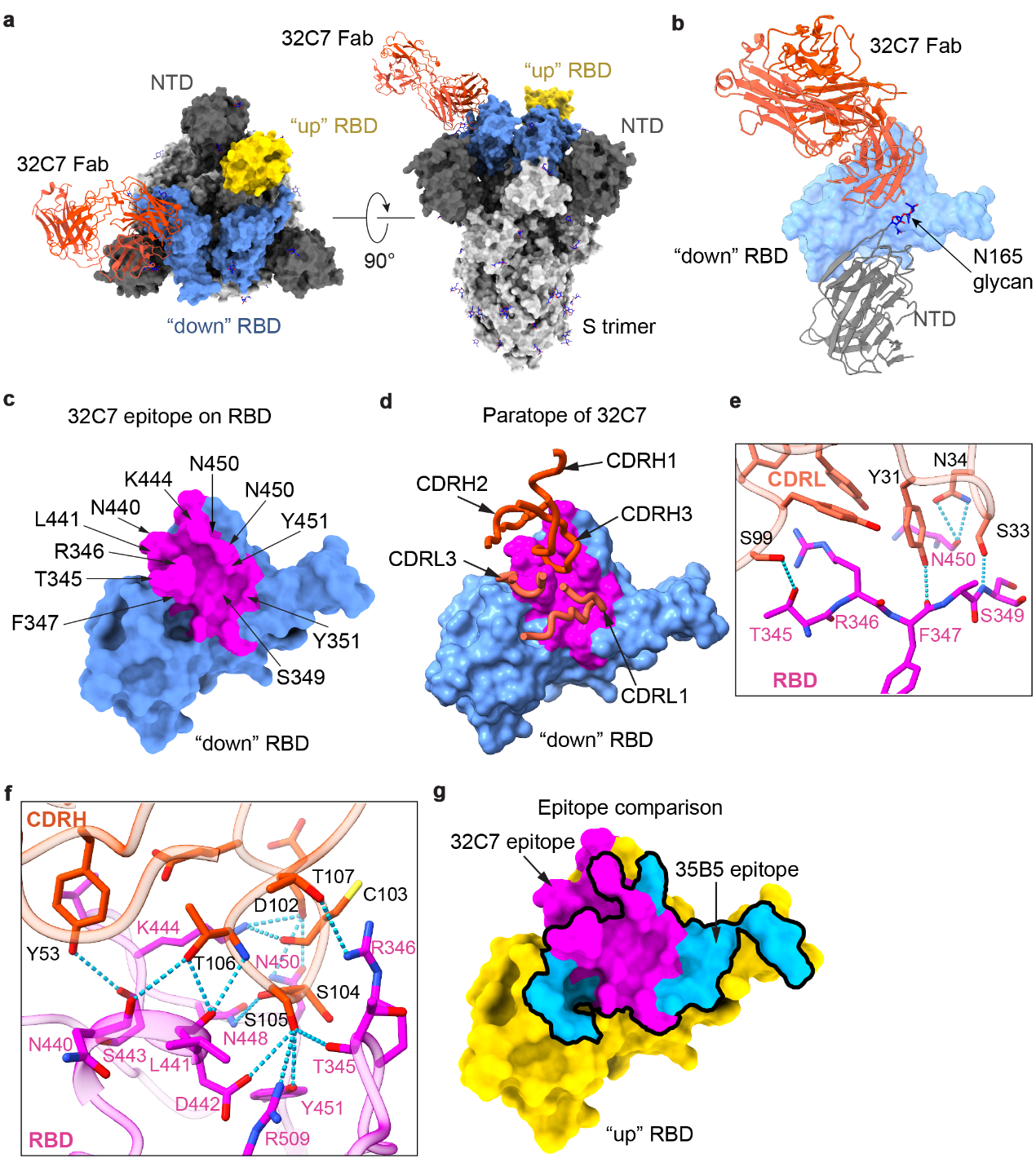


**Figure. S6. Cryo-EM structure of the S-6P-32C7 Fab complex. a** The 2.8-Å cryo-EM structure of the S-6P-32C7 Fab complex. The S-6P trimer is represented as surface. 32C7 Fab is shown in cartoon in red. The “down” and “up” RBDs are colored in blue and yellow, respectively. **b** Structural superposition of the 32C7 Fab-RBD model and the tight-closed S trimer (PDBID: 6ZB5). In the structural superimposition, 32C7 Fab does not form structural clashes with the NTD domain of the tight-closed S trimer. RBD is shown as surface in blue. The NTD domain of the S trimer is shown in cartoon in grey. **c** The 32C7 epitope on RBD. The epitope residues are labeled as indicated. **d** The RBD-interacting regions in 32C7 Fab. The CDRH1, CDRH2, CDRH3, CDRL1 and CDRL3 are shown in ribbon. **e**, **f** Detailed interactions of the light chain CDRs (**e**) and heavy chain CDRs (**f**) of 32C7 Fab with RBD. Hydrogen-bond interactions are shown as dashed lines. **g** Comparison of the epitopes for 32C7 and 35B5 on the surface of RBD. The epitopes for 32C7 and 35B5 (black contour) are colored in purple and blue, respectively.


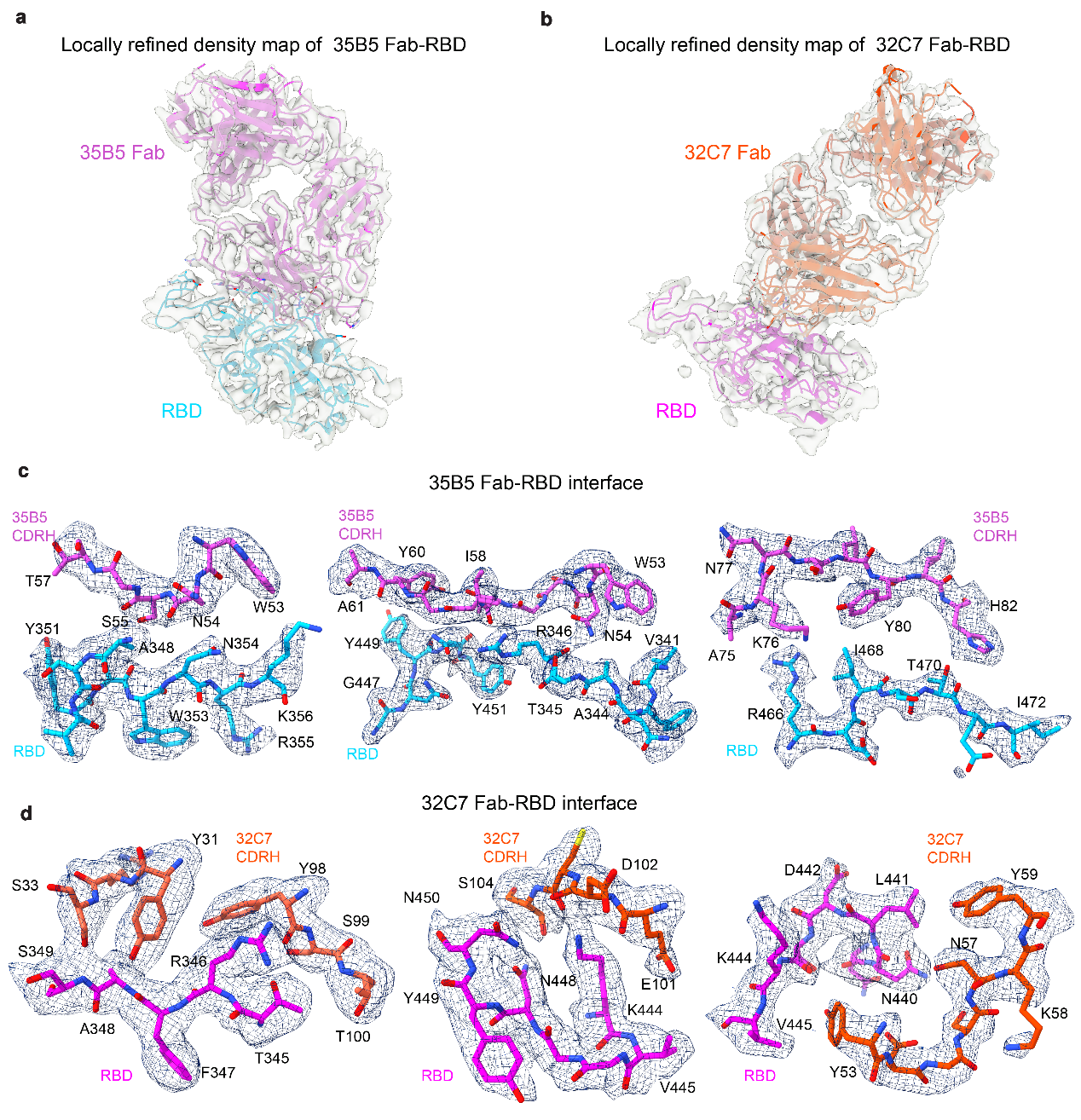


**Figure. S7. Density maps of 35B5 Fab-RBD and 32C7 Fab-RBD interfaces after local refinement. a** Locally refined density map (transparent gray) of the 35B5 Fab-“up” RBD region in the State 2 S-6P-35B5 Fab complex. **b** Locally refined density map of the 32C7 Fab-RBD region in the S-6P-32C7 Fab complex. **c** Representative density maps of interaction regions at the 35B5 Fab-RBD interface. **d** Representative density maps of interaction regions at 32C7 Fab-RBD interface. The interacting residues of RBD and Fabs are shown as stick.

|  | **35B5 Fab-S-6P**  **(state 1)** | **35B5 Fab-S-6P**  **(state 1, local refinement)** | | **35B5 Fab-S-6P**  **(state 2)** | | **35B5 Fab-S-6P**  **(state 2, local refinement)** | | **35B5 Fab-S-6P**  **(state 3)** | | **32C7 Fab-S-6P** | | **32C7 Fab-S-6P**  **(local refinement)** | |
| --- | --- | --- | --- | --- | --- | --- | --- | --- | --- | --- | --- | --- | --- |
| **PDB code** | **7E9N** | **7F46** | | **7E9O** | | **7E9P** | | **7E9Q** | | **7ENF** | | **7ENG** | |
| **EMDB code** | **EMD-31033** | **EMD-31444** | | **EMD-31034** | | **EMD-31035** | | **EMD-31036** | | **EMD-31209** | | **EMD-31210** | |
|  | | | | | | | | | | | | | |
| **Data collection and**  **processing** | | | | | | | | | | | | | |
| Voltage (kV) | 300 | | 300 | | 300 | | 300 | | 300 | | 300 | | 300 |
| Magnification | 81,000 | | 81,000 | | 81,000 | | 81,000 | | 81,000 | | 81,000 | | 81,000 |
| Pixel size (Å/pix) | 0.5475 | | 0.5475 | | 0.5475 | | 0.5475 | | 0.5475 | | 0.53865 | | 0.53865 |
| Frames per exposure | 32 | | 32 | | 32 | | 32 | | 32 | | 32 | | 32 |
| Exposure (e^-^/Å^2^) | 50 | | 50 | | 50 | | 50 | | 50 | | 50 | | 50 |
| Defocus range (μm) | 1.2 to 1.3 | | 1.2 to 1.3 | | 1.2 to 1.3 | | 1.2 to 1.3 | | 1.2 to 1.3 | | 1.4 to 1.8 | | 1.4 to 1.8 |
| Final particle images (no.) | 62,600 | | 62,600 | | 122,887 | | 122,886 | | 75,721 | | 119,062 | | 119,062 |
| Symmetry imposed | C1 | | C1 | | C1 | | C1 | | C1 | | C1 | | C1 |
| Map resolution (Å, 0.143 FSC threshold) | 3.7 | | 4.8 | | 3.4 | | 3.7 | | 3.6 | | 2.8 | | 3.6 |
| **Refinement** | | | | | | | | | | | | | |
| Map resolution (Å, 0.5 FSC threshold) | 3.9 | | 8.6 | | 3.7 | | 3.9 | | 3.8 | | 3.0 | | 3.7 |
| Map sharpening *B* factor (Å^2^) | 81.9 | | 115.6 | | 90.4 | | 86.3 | | 82.5 | | 75.8 | | 110.2 |
| Model composition | | | | | | | | | | | | | |
| Protein residues | 4,313 | | 842 | | 4338 | | 639 | | 4,338 | | 3,390 | | 640 |
| Ligands | 27 | | 2 | | 27 | | 1 | | 27 | | 53 | | 2 |
| R.m.s. deviations | | | | | | | | | | | | | |
| Bond length (Å) | 0.008 | | 0.006 | | 0.004 | | 0.004 | | 0.005 | | 0.005 | | 0.006 |
| Bond angles (º) | 0.878 | | 0.848 | | 0.768 | | 0.781 | | 0.887 | | 0.835 | | 0.802 |
| Ramachandran plot | | | | | | | | | | | | | |
| Favored (%) | 91.90 | | 91.12 | | 95.12 | | 92.53 | | 93.60 | | 94.70 | | 92.59 |
| Allowed (%) | 8.10 | | 8.88 | | 4.88 | | 7.47 | | 6.40 | | 5.30 | | 7.41 |
| Outliers (%) | 0 | | 0 | | 0 | | 0 | | 0 | | 0 | | 0 |
| Validation | | | | | | | | | | | | | |
| Poor rotamers (%) | 0.08 | | 0 | | 0.24 | | 0 | | 0.32 | | 0.56 | | 0.36 |
| Clash score | 12.44 | | 11.56 | | 10.62 | | 11.14 | | 14.62 | | 10.14 | | 14.32 |
| MolProbity score | 2.10 | | 2.09 | | 1.88 | | 2.03 | | 2.09 | | 1.89 | | 2.13 |

**Table S1. Cryo-EM data collection and refinement statistics.**
